# Multiplex preamplification of specific cDNA targets prior to gene expression analysis by TaqMan Arrays

**DOI:** 10.1186/1756-0500-1-21

**Published:** 2008-06-05

**Authors:** Lourdes Mengual, Moisès Burset, Mercedes Marín-Aguilera, María José Ribal, Antonio Alcaraz

**Affiliations:** 1 Laboratory and Department of Urology. Institut Clínic de Nefrologia i Urologia (ICNU), Hospital Clínic de Barcelona. Institut d'Investigacions Biomèdiques August Pi i Sunyer (IDIBAPS), Universitat de Barcelona, Villarroel, 170, 08036 Barcelona, Spain; 2 Molecular Biology Laboratory. Fundació Puigvert. Universitat Autònoma de Barcelona, Cartagena 340-350, 08025 Barcelona, Spain

## Abstract

**Background:**

An accurate gene expression quantification using TaqMan Arrays (TA) could be limited by the low RNA quantity obtained from some clinical samples. The novel cDNA preamplification system, the TaqMan PreAmp Master Mix kit (TPAMMK), enables a multiplex preamplification of cDNA targets and therefore, could provide a sufficient amount of specific amplicons for their posterior analysis on TA.

**Findings:**

A multiplex preamplification of 47 genes was performed in 22 samples prior to their analysis by TA, and relative gene expression levels of non-preamplified (NPA) and preamplified (PA) samples were compared. Overall, the mean cycle threshold (CT) decrement in the PA genes was 3.85 (ranging from 2.07 to 5.01). A high correlation (r) between the gene expression measurements of NPA and PA samples was found (mean r = 0.970, ranging from 0.937 to 0.994; p < 0.001 in all selected cases). High correlation coefficients between NPA and PA samples were also obtained in the analysis of genes from degraded RNA samples and/or low abundance expressed genes.

**Conclusion:**

We demonstrate that cDNA preamplification using the TPAMMK before TA analysis is a reliable approach to simultaneously measure gene expression of multiple targets in a single sample. Moreover, this procedure was validated in genes from degraded RNA samples and low abundance expressed genes. This combined methodology could have wide applications in clinical research, where scarce amounts of degraded RNA are usually obtained and several genes need to be quantified in each sample.

## Findings

## Background

TaqMan Arrays (TAs) have recently been introduced as a novel approach to measure gene expression. They combine the high sensitivity provided by the real time quantitative reverse-transcription polymerase chain reaction (qRT-PCR) with the ability to simultaneously assay RNA expression levels of up to 384 target genes in a single sample [1,2]. However, an accurate quantification with this approach could be limited by the low sample amount commonly encountered in clinical research. Therefore, it is necessary to preamplify samples to generate enough cDNA copies to enable an accurate quantification of transcripts and maintain high sensitivity.

Most of the current protocols for increasing small amounts of mRNA are designed to globally amplify all the transcriptome instead of the specific targets of interest and are usually validated by DNA array, which is a more imprecise methodology than qRT-PCR to quantify individual genes [3,4].

Recently, a novel system, the TPAMMK, to increase cDNA quantity prior to gene expression analysis by conventional quantitative PCR has been described [5-8]. However, there is no study validating this approach prior to gene expression quantification by means of TA. The possibility of simultaneously measuring gene expression of dozens to hundreds of genes in those samples that yield scarce quantities of RNA would be of great interest.

Here, we evaluate this cDNA preamplification system (TPAMMK) prior to gene expression quantification by TA. We test the approach in the context of bladder cancer detection, by analyzing RNA samples obtained from bladder fluids. Furthermore, this type of sample provides us the possibility to analyze the reliability of this combined methodology for preamplifying cDNA from degraded RNA samples and/or low abundance expressed genes.

## Results and discussion

One of the major drawbacks for the clinical use of RNA based techniques is the difficulty of obtaining sufficient quantities of high quality RNA from some human samples. The novel multiplexed miniaturized format provided by TAs could be limited by the scarce quantities of isolated RNA, since the sample is divided into a large number of aliquots [9]. Several strategies for amplifying RNA [10-12] or cDNA have been described [5-8,13,14], but to date, there is no published study validating that cDNA preamplification prior to TA analysis provides a reliable representation of gene expression profiling.

In this study, we have used the TPAMMK (AB) to preamplify 47 genes in 22 bladder fluids samples prior to their analysis by TA. This preamplification process allowed us to load a 30 fold greater quantity of cDNA in each TA port (500 ng for NPA *vs *~15000 ng for PA samples) which should theoretically result in a decrement of 4.9 CTs. However, we found an overall mean CT decrement of 3.85 (ranging from 2.07 to 5.01) in PA samples (Table 1). Although variations in amplification efficiencies (AE) of the primers/probe sets used in TA methodology have been found in this study and others [1], they do not help to explain differences between the theoretical and the experimental CT decrement described, since there is no significant correlation between the experimental CT decrement and the primers/probe set AE, neither before (r = 0.062; p = 0.678) nor after (r = 0.060; p = 0.097) the cDNA preamplification process (Table 1).

**Table 1:** Mean CT and mean AE for the 46 bladder cancer related genes and the endogenous control *GUSB *obtained before and after cDNA preamplification.

**Gene symbol**	**Primer/probe set (AB)**	**Validated samples**		**NPA samples**		**PA samples**		**Mean Ct decrement**	**r**	**NPA samples**		**PA samples**	
					
		**n NPA**	**n PA**	**Mean Ct**	**St Dev**	**Mean Ct**	**St Dev**			**Mean AE**	**St Dev**	**Mean AE**	**St Dev**
*ANK2*	Hs00153998_m1	1	18	29,22	-	25,90	-	3,32	-	1,889	-	1,794	-
*ANLN*	Hs00218803_m1	17	20	28,50	1,58	23,97	1,64	4,53	0,966	1,807	0,06	1,834	0,09
*ANXA10*	Hs00200464_m1	16	20	27,19	2,83	22,87	2,45	4,32	0,992	1,987	0,05	1,962	0,08
*ASAM*	Hs00293345_m1	3	15	29,39	1,08	25,31	0,93	4,09	0,965	1,926	-	1,752	-
*ASPM*	Hs00411505_m1	16	21	27,88	1,63	23,93	1,52	3,95	0,978	1,897	0,05	1,838	0,09
*C14orf78*	Hs00746838_s1	16	22	28,65	1,78	24,56	1,52	4,09	0,991	1,933	0,06	1,796	0,09
*CCNA2*	Hs00153138_m1	17	20	28,65	1,78	25,22	1,74	3,42	0,93	1,821	0,03	1,688	0,06
*CDC2*	Hs00364293_m1	16	20	28,47	1,77	23,86	1,74	4,61	0,96	1,867	0,06	1,823	0,09
*CDC20*	Hs00415851_g1	20	20	28,09	2,19	24,09	2,12	4,01	0,973	1,816	0,05	1,803	0,09
*CDCA1*	Hs00230097_m1	13	20	28,76	1,40	24,37	1,26	4,40	0,944	1,944	0,02	1,768	0,10
*CENPF*	Hs00193201_m1	16	21	28,20	1,65	24,55	1,58	3,65	0,96	1,898	0,04	1,840	0,10
*CFH*	Hs00164830_m1	19	22	26,66	2,40	22,71	2,15	3,95	0,978	1,832	0,04	1,786	0,02
*CRH*	Hs00174941_m1	9	11	25,42	2,67	23,01	2,31	2,40	0,967	1,836	0,06	1,711	0,11
*CTSE*	Hs00157213_m1	19	21	25,60	3,52	21,38	3,38	4,22	0,997	1,858	0,05	1,842	0,03
*CYP24A1*	Hs00167999_m1	19	21	26,40	3,11	22,62	2,93	3,78	0,994	1,944	0,07	1,940	0,08
*EBF*	Hs00395513_m1	3	14	29,87	0,67	26,27	0,44	3,60	0,817	1,665	-	1,651	-
*FGFR3*	Hs00179829_m1	18	20	23,88	2,76	21,26	2,67	2,62	0,967	1,833	0,04	1,753	0,07
*FOXM1*	Hs00153543_m1	15	20	28,38	2,00	24,67	1,88	3,71	0,971	1,896	0,05	1,718	0,05
*GJB2*	Hs00269615_s1	21	22	26,13	2,39	22,27	2,36	3,86	0,984	1,864	0,05	1,888	0,06
*GUSB*	Hs99999908_m1	22	22	25,18	2,08	20,79	2,14	4,40	-	1,914	0,04	1,942	0,07
*IGF2*	Hs00171254_m1	18	21	25,15	3,98	23,08	3,90	2,07	0,993	1,835	0,06	1,743	0,07
*IQGAP3*	Hs00603642_m1	14	20	28,29	1,52	24,18	1,49	4,11	0,978	1,886	0,03	1,782	0,04
*KIF20A*	Hs00194882_m1	12	20	28,11	1,47	24,26	1,47	3,85	0,982	1,905	0,03	1,786	0,08
*KIF2C*	Hs00199232_m1	17	21	27,97	1,87	23,88	1,93	4,09	0,966	1,804	0,05	1,739	0,06
*KIF4A*	Hs00602211_g1	13	20	28,27	1,56	24,02	1,37	4,25	0,968	1,872	0,07	1,797	0,08
*KLF9*	Hs00230918_m1	21	22	27,60	1,66	23,65	1,54	3,95	0,955	1,793	0,08	1,805	0,07
*KRT14*	Hs00265033_m1	12	20	29,12	1,34	25,66	1,54	3,45	0,97	1,995	0,08	1,780	0,15
*KRT20*	Hs00300643_m1	19	21	23,21	3,05	19,54	2,84	3,66	0,986	1,977	0,05	2,019	0,08
*MAGEA3*	Hs00366532_m1	5	8	28,01	0,44	24,50	0,73	3,51	0,935	1,790	0,05	1,747	0,03
*MAGEA9*	Hs00245619_s1	13	21	27,44	1,74	23,99	1,66	3,45	0,986	1,874	0,02	1,736	0,07
*MCM10*	Hs00218560_m1	9	20	28,82	1,09	25,16	1,12	3,66	0,968	1,861	0,03	1,694	0,03
*MELK*	Hs00207681_m1	13	20	28,33	1,58	24,83	1,60	3,50	0,932	1,821	0,07	1,671	0,10
*MMP1*	Hs00233958_m1	18	21	26,04	2,92	22,34	2,91	3,71	0,986	1,686	0,06	1,640	0,07
*MMP12*	Hs00159178_m1	12	18	28,19	2,17	23,17	2,11	5,01	0,987	1,846	0,11	1,818	0,09
*NEK2*	Hs00601227_mH	12	21	28,78	1,56	24,39	1,47	4,38	0,963	1,737	0,06	1,773	0,09
*NR2F1*	Hs00818842_m1	6	19	29,08	1,52	24,77	1,51	4,31	0,984	1,845	0,05	1,790	0,01
*PDZRN3*	Hs00392900_m1	10	22	26,72	2,07	23,11	1,74	3,61	0,99	1,888	0,05	1,736	0,11
*POLQ*	Hs00198196_m1	10	19	29,47	1,09	24,98	1,20	4,49	0,914	1,807	0,07	1,608	0,15
*POSTN*	Hs00170815_m1	4	12	28,96	1,45	24,70	1,47	4,26	0,983	1,904	0,09	1,837	0,12
*PPIA*	Hs99999904_m1	22	22	21,54	2,32	17,76	2,29	3,78	0,927	1,912	0,04	1,991	0,10
*PPP1R14D*	Hs00214613_m1	8	18	28,18	2,05	24,29	1,68	3,89	0,989	1,886	0,07	1,833	0,14
*PTPRC*	Hs00236304_m1	22	22	25,15	2,03	21,62	2,18	3,53	0,986	1,944	0,06	1,886	0,09
*SLC1A6*	Hs00192604_m1	7	12	27,54	2,05	23,63	1,34	3,92	0,978	1,743	0,03	1,683	0,02
*TERT*	Hs00162669_m1	2	12	29,40	1,13	26,10	1,64	3,31	-	1,856	-	1,697	-
*TOP2A*	Hs00172214_m1	19	20	27,40	2,42	23,31	2,27	4,09	0,909	1,890	0,06	1,873	0,09
*TPX2*	Hs00201616_m1	18	20	27,69	2,17	23,91	2,04	3,78	0,968	1,990	0,07	1,931	0,07
*TRIP13*	Hs00188500_m1	18	20	28,03	1,96	23,83	1,90	4,21	0,976	1,893	0,05	1,806	0,05

**Key: **CT, Cycle Threshold; AE, Amplification Efficiency; NPA, Non-Preamplified; PA, Preamplified; St Dev, Standard Deviation.

On the other hand, linear regression analysis showed a high correlation (r) between gene expression measurements of NPA and PA samples for the validated genes (mean r values = 0.970, ranging from 0.937 to 0.994, p < 0.001 in all cases, except for sample number 15 where only 3 genes could be analyzed) (Table 2). Thus, albeit CT decrement for each individual gene is not uniform, the overall relative gene expression levels in PA samples remained proportional to the original gene expression levels in NPA samples. However, when checking preamplification uniformity individually for each gene (ΔΔCT values within ± 1.5) we found that there were three genes (*IGF2*, *FGFR3 *and *CRH*) that were consistently non-uniformly preamplified in a significant number of samples and therefore were not suitable for preamplification (Table 2). We do not have a clear explanation for this fact, although transcript abundance, primers and probe localization or amplicon length/sequence could influence the preamplification efficiency. From these results, it becomes clear that cDNA preamplification before gene expression quantification by TA can facilitate the analysis of multiple target genes from very low quantities of RNA in a single experiment. However, checking the preamplification uniformity in each target gene with control material before its evaluation in testing samples is mandatory.

**Table 2:** Summary of the clinical characteristics, RNA quality and cDNA preamplification results for the 22 samples analyzed in this study.

**Sample number**	**Type of sample**	**Type of bladder fluid**	**Tumor characteristics**		**RNA characteristics**				**N° validated genes (CT ≤ 31)**		**Correlation coeficients (r)**	**N° validated genes in NPA samples with ΔΔCT outside ± 1.5**	**Gene symbol**
										
					**rRNA ratio**	**RIN**	**% of total**						
									
			**stage**	**grade**			**18S area**	**28S area**	**NPA samples**	**PA samples**			
1	T	BW	Ta	LG	2	9.8	20.6	40.4	33	43	0.986	1	*IGF2*
2	T	BW	Ta	LG	1.7	9.2	18.5	31.6	39	46	0.978	4	*CRH, FGFR3, IGF2,KRT14*
3	T	BW	Ta	LG	1.7	8.9	18.5	32.1	38	45	0.987	2	*CRH,IGF2*
4	T	BW	Ta	LG	1.6	8.8	17.0	26.8	36	45	0.991	1	*IGF2*
5	T	BW	Ta	LG	1.3	8.2	14.2	18.1	25	42	0.987	1	*IGF2*
6	T	BW	T2b+CIS	HG	1.8	8	9.3	16.7	38	44	0.967	3	*CCNA2, IGF2, MAGEA9*
7	T	BW	T1	HG	2.3	7.8	8.1	18.7	35	43	0.975	3	*FGFR3, IGF2, MELK*
8	C	BW	-	-	1.5	7.6	8.6	12.6	19	40	0.985	0	-
9	T	BW	T1c	HG	1.6	7.5	9.4	15.0	32	42	0.98	5	*FGFR3, IGF2, KIF2C, MELK, PDZRN3*
10	T	U	T1c+CIS	HG	1.1	7.5	13.6	15.0	40	45	0.961	2	*CRH, IGF2*
11	T	U	T1b	HG	0.8	7.5	12.8	10.1	41	46	0.956	3	*CRH, FGFR3, IGF2*
12	T	U	T1	HG	1.2	7.4	10.5	12.3	39	45	0.972	2	*CRH, IGF2*
13	C	BW	-	-	1.3	7.2	7.9	10.0	28	39	0.976	1	*IGF2*
14	T	BW	T1+CIS	HG	1.3	6.4	6.4	8.4	35	43	0.975	3	*CRH, FGFR3, IGF2*
15	C	BW	-	-	0.8	2.8	1,6	1,3	3	13	0.937	0	_
16	C	U	-	-	0	2.5	N/A	N/A	8	14	0.95	1	*KRT14*
17	T	U	T2	HG	0	2.5	N/A	N/A	11	39	0.994	1	*CDC2*
18	T	U	T1	HG	0	2.4	N/A	N/A	13	41	0.965	3	*GJB2, FGFR3, IGF2*
19	T	U	T2+CIS	HG	0	2.4	N/A	N/A	27	40	0.97	5	*CCNA2, CRH, FGFR3, IGF2, KRT14*
20	T	U	T2+CIS	HG	0	2.3	N/A	N/A	31	45	0.938	2	*IGF2, TOP2A*
21	C	BW	-	-	1.3	N/A	3.8	4.6	18	36	0.943	2	*IGF2, FGFR3*
22	T	BW	T2b	HG	1.1	N/A	8.6	9.4	40	45	0.968	1	*IGF2*

**Legend/key: **Samples are ranked by RIN number. BW, Bladder Washing; CIS, Carcinoma In Situ; C, Control; CT, Cycle Threshold; HG, High Grade; LG, Low Grade; N/A, Non Available; NPA, Non-Preamplified; PA, Preamplified; RIN, RNA Integrity Number; T, Tumor; U, Urine.

As the described CT decrement would not be sufficient to accurately quantify gene expression in some cases, it must be mentioned that, according to kit manufacturers, it is possible to perform up to 14 preamplification cycles, as well as to increase the quantity of cDNA in the preamplification reaction up to 250 ng or to load more preamplified volume of cDNA into each TA port to achieve the desired decrement.

Since our goal was to test the suitability of the cDNA preamplification of specific target genes before their quantification by TA, we have only analyzed those genes with a reliable CT value in NPA samples (CT ≥ 31). However, to determine the linearity of the cDNA preamplification in low abundant expressed genes (those target genes with a CT value > 31 in NPA samples), we performed two serial dilutions (1/20 and 1/400) of 3 cDNAs from NPA samples with different RNA degradation levels (sample number 2, 11 and 22) and preamplified them with the same protocol used for the non diluted NPA samples. Subsequently, we compared the ΔCT values from diluted PA samples with the corresponding ΔCT of the initial cDNA (non diluted NPA) samples. This comparison yielded correlation coefficients close to 1, indicating that the vast majority of low abundant expressed genes are correctly preamplified and that the preamplification process maintains relative gene expression levels of the initial RNA over a broad range of CT values (Table 3).

**Table 3:** Number of validated genes of two serial dilutions of 3 cDNA samples before and after their preamplification.

	**Initial cDNA**			
	
	**25 ng of preamplified cDNA**			
Sample number	N° validated genes (CT ≤ 31)		N° validated genes with ΔΔCT outside ± 1.5	r
			
	NPA samples	PA samples		

2	39	46	4	0.978
11	41	46	3	0.956
22	40	45	1	0.968

***ng cDNA/port***	***500***	***15000***		

**Dilution 1/20 of the initial cDNA**				

	**1.25 ng of preamplified cDNA**			

Sample number	N° validated genes (CT ≤ 31)		N° validated genes with ΔΔCT outside ± 1.5	r
			
	NPA samples	PA samples		

2	14	38	6	0.972
11	22	40	2	0.967
22	6	39	5	0.988

***ng cDNA/port***	***25***	***750***		

**Dilution 1/400 of the initial cDNA**				

	**0.06 ng of preamplified cDNA**			

Sample number	N° validated genes (CT ≤ 31)		N° validated genes with ΔΔCT outside ± 1.5	r
			
	NPA samples	PA samples		

2	3	17	0	0.962
11	2	27	3	0.883
22	3	20	2	0.932

***ng cDNA/port***	***1.25***	***37.5***		

**Legend/key: **The number of each sample corresponds to the "sample number" indicated in Table 2. CT, Cycle Threshold; NPA, Non-Preamplified; PA, Preamplified.

For some time, it has been believed that degraded RNA samples were not suitable for gene expression studies. Nevertheless, many authors have recently reported the use of this material for gene profiling using DNA microarrays as well as qRT-PCR approaches [12,15]. In order to investigate whether RNA degradation influences the efficiency of preamplification, gene expression measurements from those samples with a high RNA quality (RIN > 8; n= 6), those with good RNA quality (5 < RIN < 8; n= 8) and those with low RNA quality (RIN < 5; n= 6) [16] were compared (Table 2). The two samples with non available RIN were excluded from this part of the study. The mean CT decrement after preamplification was very similar in the three groups of samples; 3.90, 3.68 and 4.00, respectively. As expected, we initially found that the average number of validated genes corresponds to the RNA integrity (35.8, 34.6 and 16.5 in high, good and low RNA quality samples, respectively). The average number of validated genes in the three groups of samples after the cDNA preamplification was 45.2, 43.9 and 32.8 in high, good and low RNA quality samples, respectively, resulting from the increment in the number of validated genes being much higher in degraded RNA than from high/good quality RNA samples.

Thus, we have been able to demonstrate that preamplifed cDNA from samples with different RNA degradation states is a suitable material for TA analysis, facilitating the simultaneous analysis of multiple targets in a single experiment from archived pathology specimens in retrospective studies.

## Conclusion

To our knowledge, this is the first quantitative gene expression report validating that cDNA preamplification using the TPAMMK prior to TA analysis preserves relative transcript expression levels of individual genes. The possibility to increase cDNA quantity before its analysis by TA opens up the possibility of analysing multiple target genes in a single experiment in those samples that yield scarce quantities of RNA. Furthermore, this approach is suitable for preamplifying genes from degraded RNA and low abundance expressed genes. This combined methodology could have wide applications in clinical research, where scarce amounts of degraded RNA are usually obtained and several genes needs to be quantified in each sample.

## Methods

### Patients and samples

Ten bladder washings (BW) and 7 voided urine specimens from patients with pathologically diagnosed bladder cancer (BC) [17,18] and 4 BW and 1 urine sample from patients without history of BC (controls) were collected between April 2004 and September 2005 after informed consent (Table 2).

Ice cooled BW or urine samples were mixed with 1/25 volumes of 0.5 M EDTA, pH 8.0 and were centrifuged at 1000 × g for 10 minutes. The cell pellets were re-suspended in 1 ml of TRIzol reagent (Invitrogen, Carlsbad, CA, USA) and frozen at -80°C until RNA extraction.

### RNA extraction and cDNA synthesis

RNAs were extracted using TRIzol reagent (Invitrogen, Carlsbad, CA, USA) according to manufacturer's instructions. Total RNA was quantified by spectrophotometric analysis at 260 nm and RNA degradation was assessed using the Agilent 2100 Bioanalyzer (Agilent Technologies, Waldbronn, Germany) [19] (Table 2).

cDNA was synthesized from 1 μg of total RNA using the High Capacity cDNA Archive Kit (Applied Biosystems, Foster City, USA, hereafter referred as AB) following manufacturer instructions, except that the final volume of the reaction was 50 μl.

### Multiplex preamplification of cDNA targets

**Figure 1:**
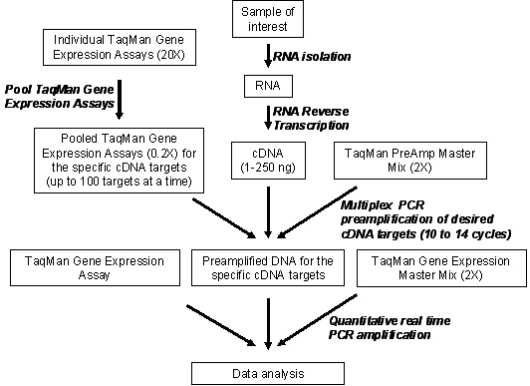
**Workflow for the entire preamplification process**. Detailed steps are described in the Material and Methods section. Briefly, to increase the quantity of the specific cDNA targets for gene expression analysis using TaqMan methodology, cDNA from the reverse transcription is mixed with pooled TaqMan Gene Expression Assays and with TaqMan PreAmp MasterMix. After the multiplex preamplification of desired cDNA targets, quantitative real time PCR amplification of preamplified target cDNAs, using sequence-specific primers and TaqMan probes from the TaqMan Gene Expression Assays and TaqMan Gene Expression Master Mix, is performed.

A multiplex PCR preamplification of the 46 specific cDNA targets and the endogenous control *GUSB *(Table 1) was performed using TPAMMK following manufacturer's instructions (AB). The 47 TaqMan Gene Expression Assays (AB) were pooled together at 0.2× final concentration. Subsequently, 12.5 μl of the pooled assay mix (0.2X) were combined with 25 ng of each cDNA sample and 25 μl of the TaqMan PreAmp Master Mix (2X) in a final volume of 50 μl (Figure 1). Thermal cycling conditions were as follows: initial hold at 95°C during 10 min and ten preamplification cycles of 15 sec at 95°C and 4 min at 60°C.

For samples n° 2, 11 and 22, two dilutions (1/20 and 1/400) of the NPA cDNA were prepared and 1.25 μl of each cDNA dilution (1.25 ng and 0.0625 ng, respectively) were subsequently preamplified with the same protocol described above.

### TaqMan Arrays (TA)

The NPA and PA target cDNAs were then amplified in singleplex reactions using TA following manufacturer's recommendations (AB). Commercially available TaqMan Gene Expression Assays (AB) for all the 46 genes differentially expressed in bladder cancer specimens (data not shown) and the endogenous control *GUSB *were used (Table 1). Twenty-five μl of NPA cDNAs and 30 μl of PA cDNAs were mixed with 50 μl of 2× TaqMan Universal PCR Master Mix (AB) in a final volume of 100 μl. After loading mixes into the TA ports, cards were centrifuged twice for 1 min at 1200 rpm, sealed and run in an ABI PRISM 7900HT SDS with the following thermal conditions: 2 min at 50°C, 10 min at 94.5°C, 40 cycles of denaturation at 97°C for 30 sec and annealing and extension at 59.7°C for 1 min.

### Data analysis

Quantitative real time PCR data were processed with SDS 2.1 software package (AB). A defined baseline of 3 to 12 cycles and a defined threshold of 0.35 were used for all the genes to record the cycle thresholds (CTs). Since precision on TA starts dropping off at around 30–32 CTs, assays that yielded a CT > 31 cycles were excluded from the analysis and comparisons between PA and NPA genes were performed only taking into account those genes with a CT value ≤ 31 in NPA samples (named validated genes). Data normalization was carried out with reference gene *GUSB*.

Linear regression analysis was performed to compare gene expression data (ΔCT) from NPA targets (ΔCT_NPA _= CT_NPA target _-CT_NPA *GUSB*_) *vs *PA targets (ΔCT_PA _= CT_PA target _-CT_NPA *GUSB*_). Those regressions with less than 4 points were excluded from the analysis. Specific gene preamplification uniformity was checked calculating the ΔCT_NPA _and ΔCT_PA_, and determining the ΔΔCT between NPA and PA targets (ΔΔCT = ΔCT_PA _-ΔCT_NPA_). ΔΔCT values close to zero indicated preamplification uniformity. Targets that produce ΔΔCT values within ± 1.5 were considered uniformly preamplified (TPAMMK Protocol, AB). AE of each assay was calculated from fluorescent data using the DART-PCR software version 1.0 [20,21]. Pfaffl (2001) definition for AE has been used [22].

## Competing interests

The authors declare that they have no competing interests.

## Authors' contributions

All authors participated in study concept, design and interpretation of data. LM carried out the experiments, participated in acquisition and analysis of data and drafting of the manuscript. MB participated in analysis of data, provided statistical expertise and critical revision of the manuscript. MM-A participated in collection of samples, assisted some experiments and critical revision of the manuscript. MJR participated in collection of samples, provided clinical expertise and critical revision of the manuscript. AA provided clinical expertise, critical revision of the manuscript, obtained funding and supervised conduct of the study. All the authors have read and approved the final manuscript.
